# Bridging the Gap between Basic Research and Clinical Practice: The Growing Role of Translational Neurorehabilitation

**DOI:** 10.3390/medicines10080045

**Published:** 2023-08-01

**Authors:** Mirjam Bonanno, Rocco Salvatore Calabrò

**Affiliations:** IRCCS Centro Neurolesi “Bonino-Pulejox”, Via Palermo, SS 113, C. da Casazza, 98124 Messina, Italy; mirjam.bonanno@irccsme.it

**Keywords:** translational neuroscience, translational neurorehabilitation, neurological disorders

## Abstract

Translational neuroscience is intended as a holistic approach in the field of brain disorders, starting from the basic research of cerebral morphology and with the function of implementing it into clinical practice. This concept can be applied to the rehabilitation field to promote promising results that positively influence the patient’s quality of life. The last decades have seen great scientific and technological improvements in the field of neurorehabilitation. In this paper, we discuss the main issues related to translational neurorehabilitation, from basic research to current clinical practice, and we also suggest possible future scenarios.

## 1. Introduction

**Figure 1:**
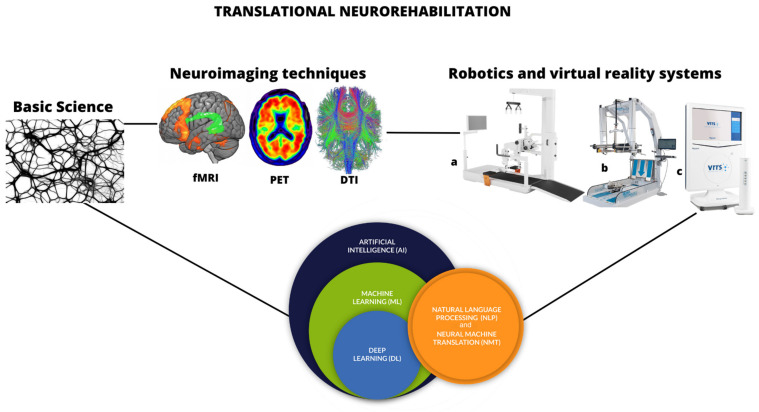
Theoretical paradigm about translational neurorehabilitation and its components, starting from basic science to arrive at robotic and virtual reality devices ((**a**): Lokomat (exoskeleton); (**b**): GE-O system (end-effector); (**c**): Virtual Reality Rehabilitation System (VRRS).

Translational neuroscience is intended as a holistic approach in the field of brain disorders, from the basic research of cerebral morphology and with the function of being implemented into clinical practice [1]. This concept can be applied to the rehabilitation field in order to promote promising results that positively influence the patient’s quality of life [2]. In detail, robotic and virtual reality devices represent the best example of translational neurorehabilitation in the last decade, according to the basic principles for motor recovery and neuroplasticity in neurological disorders (i.e., high repetition and intensity, long-lasting sessions, motivation, and multisensorial feedbacks) [3,4]. However, such developments in clinical practice would not exist without considering the role of basic research in this field. After brain damage, neural reorganization can occur through neuroplastic processes, thus promoting functional recovery [5]. At the cellular level, neural plasticity plays a pivotal role in maintaining neural connections. In particular, synaptic plasticity promotes functional and then structural changes in synaptic connections, dendric spines, and axonal modifications [6]. In this vein, the lessons learned from basic research allowed a great enrichment for neurorehabilitation practice, especially through the implementation of technological tools and systems, such as robotics and virtual reality, which are nowadays known to boost neuroplasticity [7,8]. Neuroplastic phenomena can be monitored through advanced neuroimaging techniques, such as non-invasive diagnostic procedures, during the neurorehabilitation path, since they are not only instruments to “describe” brain damage [9]. The combination of clinical markers and the application of multimodal neuroimaging techniques are paving the way to a new frontier in the therapeutic approach for neurological patients, also using nano agents as nanoprobes [10,11,12]. In this vein, the neurorehabilitation translational context also has been recently extended to the bioengineering field, considering the role of artificial intelligence and machine learning models used to predict motor/cognitive outcomes, also in neurological patients. In fact, there is a need to objectively quantify the progress achieved with conventional and/or advanced rehabilitation training. For example, the classic observational analysis of gait is not more reliable for both clinical and research purposes. In this sense, some authors [13,14] proposed different smart and user-friendly technologies to perform an objective gait analysis in neurological patients, including mobile devices and low-cost wearable sensors [15]. The data extracted from these tools can be used to train machine learning algorithms to differentiate patients that are at risk of falling from those who are not, promoting tailored rehabilitation training to prevent secondary complications due to falls and imbalances. This is why the “translational neuroscience” concept, which involves several disciplines (i.e., biomechanics, engineering, neurology, physiology, physiotherapy, neuropsychology, biochemistry, physical and rehabilitation medicine), can be seen as a pragmatic model given that it is strictly linked with neurorehabilitation and clinical practice (Figure 1).

## 2. Back to Basic Research: The Primary Stone of Translational Neurorehabilitation

Translational neuroscientific research needs a solid understanding of the underlying mechanisms and circuits involved in normal cerebral functions [16]. In this way, only basic research, which can be seen as a “primary stone” of clinical research, is equipped to investigate this issue. For example, a wide range of genetic studies have demonstrated how the presence of different variants of genes can increase the risk of developing multiple neurological and psychiatric disorders (i.e., Parkinson’s disease, multiple sclerosis, Alzheimer’s disease, and also autism and schizophrenia) [17]. In addition, the discovery of growth factors implicated in neuroplastic processes, such as the brain-derivate-neurotrophic factor (BDNF), influenced the developments of rehabilitation technologies [18]. The participation of BDNF in neuroplasticity is important in inducing functional and structural changes since it positively modulates the synthesis of proteins involved in synaptic changes [19]. Notably, the presence of BDNF is considered as a sign of neuroprotection and can be sustained by performing physical activity. In fact, it has been demonstrated that physical activity can significantly improve the levels of BDNF and irisin, which are involved in motor and cognitive restoring, in both humans and animals. In rats, physical training performed for 11 weeks was associated with cognitive improvements, especially in memory, in addition to neural death prevention and the promotion of neurogenesis. In a similar way, a scheduled 12- or 16-week training program in humans increased the cerebral blood flow and the production of growth factors, including BDNF [20,21]. Therefore, genetic polymorphisms involved in the production of neurotrophic factors like BDNF could have an impact on motor recovery. Moreover, the well-known polymorphism in BDNF gene (rs6265) causes valine to methionine substitution (Val66Met), which has a role in influencing memory and motor learning, in both healthy subjects and neurodegenerative diseases. According to Giordano et al. [22], the Val66Met plays a protective role toward grey matter atrophy in people affected by multiple sclerosis, who therefore potentially experience more improvements in motor recovery. Indeed, in people with stroke, basic research advanced the neurotherapeutic field with a sequential release of biomolecules focused on repair modalities (i.e., synaptic plasticity or neurogenesis), or other therapies involved in the limitation of the extent of brain damage, reducing the inflammatory response [23]. Other advancements, such as the local transport of protein therapeutics and cells, can be a promising strategy to promote functional repair [24]. From a future perspective, it would be interesting if researchers combined the use of biomaterials-based treatment and rehabilitation to achieve better and long-term outcomes.

## 3. The Role of Neuroimaging in the Neurorehabilitation Context

After brain damage, little portions of neural tissue spontaneously tend to reorganize through neuroplastic processes [25]. Neuroimaging techniques, such as functional magnetic resonance imaging (fMRI) and magnetoencephalography allows for studying these phenomena [26]. In particular, fMRI evaluates the activity of different brain regions by measuring changes in blood flow to other brain areas. Differently from PET (in which a radioactive tracer is injected), fMRI uses a strong magnetic field that can be distorted by a molecule called deoxyhaemoglobin, indicating greater brain activity [27]. Moreover, to anatomically investigate pre–post treatment neural pathways, both diffusion tensor imaging (DTI), i.e., an MRI technique that uses anisotropic diffusion to estimate the axonal (white matter) organization of the brain, and fibre tractography (FT), a 3D reconstruction technique to assess neural tracts using data collected by diffusion tensor imaging [28], can be used.

For the registration of the spontaneous electrical cerebral activity, electroencephalography is considered as a “gold standard”, especially for the diagnosis of epilepsy [29]. In fact, the EEG allows for a non-invasive recording of cerebral waves, with sets of electrodes on the patient’s scalp [30]. Indeed, EEG can be registered during the execution of specific tasks or the activity of daily living through wearable and wireless systems, using headsets and sensors [31]. With the recent technological development, the “wearable” characteristic of diagnostic devices has allowed clinicians to acquire data continuously and unobtrusively, while patients are performing their daily life activities. In addition, collecting data remotely can be used to promote independent rehabilitation training, outside the clinics. In fact, wearable sensors, during rehabilitation sessions, can reduce the evaluation times, providing more objective data than the observational scales or tests when applied alone. Potentially, these systems could reduce diagnostic errors, ensuring the most accurate treatment for each patient.

This clinical–radiological issue needs to be addressed in both clinical and neuroscientific contexts in order to further understand new patterns of brain activity. From this point of view, advanced neuroimaging could appear as a mere “descriptive” practice since it investigates the brain’s structures and functions [32]. However, recent evidence used neuroimaging techniques to individuate biological markers or for treating brain disease [33]. In this light, the approach must be integrative and multidisciplinary from the perspective of including the clinical-oriented collaboration between researchers and neuroscientists of different areas. From a translational neuroimaging research point of view, the knowledge of physiology and pathology in the human and animal neural system will further allow for the understanding of mechanisms of functional recovery after brain damage.

## 4. Innovative Technologies in Neurorehabilitation

Robotics and virtual reality (VR) devices are considered the most promising tools in the neurorehabilitation field. In detail, robotic devices can reproduce accurately human kinematics, aiding patients to perform those movements that are reduced or lost due to brain or spinal cord damage. Indeed, robotic devices can also quantify and monitor patients’ improvements objectively in order to promote a personalized and tailored rehabilitation approach [34,35]. Commonly, robotic devices are distinguished into two main categories, based on biomechanical features: exoskeletons (patients’ limbs are allocated in a robotic orthosis that allows them to move on a treadmill or over ground) and end-effectors (consisting of stationary devices in which the distal part is the only one able to move) [36]. It has been demonstrated that gait robotic rehabilitation can lead to a better outcome than conventional physiotherapy, especially in patients that are more severely affected and within the first months from the injury, independently from the device used to train the motor disability [37]. In addition, motor evaluation provided by robotic devices can be considered more objective and accurate than the observational scales or tests are, especially during the locomotion. The registration of kinematic and spatial–temporal parameters of movements allows the therapists to personalize each rehabilitation session. Moreover, the registration of these parameters becomes extremely important in the research field to understand the underlying motor mechanisms of a particular neurological condition [38]. This, in turn, allows for the training of specific machine learning algorithms to recognize altered patterns of gait, maybe before the onset of the illness itself.

Recent developments in the field have led to the production of “soft robots” that are made of compliant and/or flexible materials, including silicone, rubber, or gel that are able to bend, twist, and deform, imitating living organisms [39]. From a translational perspective, these devices were adapted to be worn by neurological patients as they can mimic the physiological movement of the body in which they are placed, in order to help patients in restoring motor functions. In this way, patients who are affected by a spinal cord injury (SCI) can use them both for upper and lower limbs during rehabilitation sessions and the activities of daily living, decreasing their fatigue and metabolic costs thanks to lighter materials than the old exoskeletons [40]. For example, the Mollii electrosuit is equipped with electrodes to deliver electrical stimulation to the muscles in order to reduce spasticity and increase flexibility and the range of motion [41]. Perpetuini et al. [40] investigated the effectiveness of Mollii electrosuits in neurological disorders, revealing that their benefits depend on the duration of administration and dosage of the treatment. However, it is worth noting that there are still no shared protocols on the dosage and timing of this electrosuit in neurological patients.

Another promising approach is the use of VR, which consists of a multisensory and interactive simulation of real-life scenarios. In this way, patients can experiment with two types of perceptive conceptions, including immersion and presence. Immersion is defined as the objective perception of a sense of “sensor absorption”, whereas “presence” is a subjective psychological state in which the user is consciously involved in the virtual environment [42]. Indeed, the VR can be combined with robotic devices during rehabilitation sessions to further promote the effects of rehabilitation training, boosting use-dependent plasticity according to the increased motivation, high rates of repetition, and intensity. Neurological patients receiving VR may achieve better results than those submitted to conventional treatments in both motor and cognitive outcomes [43,44,45].

VR has been used also at home through the so-called “telerehabilitation”, which provides the possibility to train patients remotely, thus overcoming geographic barriers, thanks to the support of tablets or mobile platforms [46,47]. This innovative method allows clinicians to follow and monitor patients directly at home, also involving their caregivers who act as co-therapists.

Recently, a new neurorehabilitation approach has spread, consisting of the combined use of neuromodulation with robotic or VR devices [8]. Non-invasive brain stimulation (NIBS) or neuromodulation techniques, including repetitive transcranial magnetic stimulation (rTMS) and transcranial direct current stimulation (tDCS), can be used to enhance post-injury recovery [48]. In fact, NIBS modulates the central nervous system by activating neurons in the brain and cortical excitability in order to further potentiate neuroplastic processes during rehabilitation training [49]. In particular, the combined use of VR and rTMS could improve the effects of rehabilitation by modulating the activity of damaged brain circuits to provide a beneficial effect [50]. This is why the integrated use of neuromodulation and the ecological virtual environment can lead to more benefits than the VR intervention alone.

Deep brain stimulation (DBS) of the internal globus pallidus (GPi) and the subthalamic nucleus (STN) were found to be effective and safe targets in patients affected by Parkinson’s disease (PD) [51]. From the translational neurorehabilitation view, DBS is also used in patients with tremors, dystonia, epilepsy, depression, Tourette’s syndrome, and obsessive-compulsive disorder, and it is generally delivered in an open-loop mode (i.e., pre-programming “on” and “off” periods). However, DBS in closed-loop mode (or adaptively) could increase the therapeutic window and reduce the power drain on the battery of the pulse generator [52]. In fact, applications in the neuromodulation field include the closed-loop neuromodulation (CLN) system. The CNL system allows the production of stimulation that can be delivered when specific physiological states or conditions occur. In addition, the parameters of the stimulation can be adjusted dynamically to optimize the effect of stimulation in real time (adaptive neurostimulation). In this way, NeuroPace (Mountain View, CA, USA) has developed a CNL device for the treatment of refractory epilepsy to elicit a pre-programmed normalizing burst of stimulation, preventing the seizure onset [53].

## 5. Artificial Intelligence and Machine Learning in the Neurorehabilitation

Artificial intelligence (AI) and machine learning (ML) are both components of computer science [54]. Besides their use in bioengineering, they can be included in the wide concept of translational neurorehabilitation. Specifically, AI allows for the creation of systems that can imitate human intelligence while ML techniques are used to extract information from a wide range of data [55]. Despite the technical issues, it is fundamental to combine clinical knowledge with these technological tools to explore their potentiality in the context of neurorehabilitation. For example, AI is yet to be used in the decision-making process, the adaptation of exercises during telerehabilitation, and the monitoring progress through the objective assessment parameters and validated scores [56]. On the other hand, ML could aid clinicians in recognizing specific motor tasks (i.e., facial expressions, walking, sitting, and so on) and in measuring treatment outcomes based on the patients’ activity performed in the real world [57]. At the molecular level, the application of ML techniques may include mechanistic-based phenotyping models focused on the prevention and treatment of mood and cognitive disorders [58]. In fact, the development of bioinformatics and ML tools have improved the knowledge about the underlying neurobiological substrates of neurological disorders [59,60,61]. This, in turn, can be translated into clinical practice for pathology classification and treatment issues. In this vein, Zhang et al. [62] developed a treatment-predictive EEG signature using ML techniques for military veterans with post-traumatic stress disorder. The findings provided by authors address the importance in the biological definition of patients with post-traumatic stress disorders that are resistant to psychotherapy.

Other interesting ML approaches include movement classification, which aims to assess the quality of motor performance during exercise training, and predictive models to foresee the patients’ functional status [63]. To this aim, Slemenšek et al. [64] tested a combination of different ML algorithms to find a robust gait motion data acquisition system that allows for either the classification of recorded gait data during the activities of daily living, or the identification of common risk factors. These findings showed that convolutional and recurrent neural networks algorithms are valid systems for detecting anomalies during gait in patients with neurological conditions, such as PD. However, the real challenge of AI applications consists of the integration of clinical practice and technical issues in assisting patients and their caregivers in a minimally supervised but more efficient way.

It is noteworthy that other approaches, such as system dynamics, may play a pivotal role in the global comprehension of the main neurological diseases including Alzheimer’s, PD, multiple sclerosis, and traumatic brain injury [65]. System dynamics is a branch of the complex systems science, and it allows for the construction of models with a non-linear relationship that produce non-linear behaviours through diagrams or simulations [65,66]. Specifically, the system dynamics approaches range from high speculative mathematical models to diagram-based and group modelling methods. These models aim to go deeper into the knowledge regarding specific mechanisms and processes involved in the patient recovery. Kenzie et al. [66] described some system dynamics models implicated in the mechanisms of traumatic brain injury and its recovery. Notably, the system dynamic regarding the comprehension of acute mechanisms of traumatic brain injuries should take into account the high variability within and between subjects. Indeed, biological diversity (with regard to genetic variations) needs also a special mention, since it can be considered as a fundamental and prognostic factor for functional recovery [67].

## 6. Current Limitations in Translational Neurorehabilitation

Both robotic-based interventions and neurorehabilitation-based biomarkers have clinical, translational, and knowledge limitations, which deeply affect their implementation in clinical practice. Clinical limitations are mainly related to studies with a small sample size, and the inter- and intra-reliability of clinical scales. In fact, more efforts in ensuring larger patient populations with a quantitative assessment should be performed, relying on robot-based evaluation or wearable sensors [68]. However, the high costs and the complexity of technology equipment may discourage their implementation in clinical practice (translational limitation). Nevertheless, it should be considered that the robotic-based neurorehabilitation approach has been shown to provide better outcomes in patients affected by severe disabilities than conventional treatments. In addition, new economic materials are being developed to reduce the cost of technology, ensuring training efficacy [69].

Moreover, technological developments and the discovery of biomarkers involved in physical recovery could define how patients are trained and how their assessment is quantified, since there is a lack of evidence about motor function and recovery mechanisms (knowledge limitation). At this point, a transdisciplinary approach to coordinate evidence could fill the gap in the “knowledge limitation” in order to provide more useful information. In fact, translational research also includes cross-disciplinary communication promoting information exchange among researchers, in order to ask better scientific questions [70]. In addition, the transdisciplinary approach could also promote non-categorical thinking, which allows one to go outside the box and explore alternative solutions to problems. In fact, categorical thinking can be considered as the natural and inevitable tendency of the human mind. Sometimes categorical thinking can limit the creative mind process, if it is based on inattention to details.

## 7. Conclusions

Translational neurorehabilitation is a growing field, that, with the support provided by innovative technologies, allows one to perform personalized treatments for patients with neurological disorders, according to the neuroplasticity and reinforcement learning principles. However, the idea of a patient-tailored approach is not new, since different types of algorithms have been proposed based on patients’ characteristics (including clinical history, time since injury, localization of the brain lesion, and functional impairment). From this point of view, biomarkers and clinical findings using objective instruments (such as gait analysis, neuroimaging and electrophysiological instruments) could support ML and AI systems not only for the prediction of specific outcomes but also to guarantee the best rehabilitation treatment. In the near future, using this promising translation approach, it will be necessary to understand who may benefit the most from this advanced technology, in order to achieve a tailored and effective treatment for neurological patients.

## Data Availability

Not applicable.

## References

[1] Davies C., Hamilton O.K.L., Hooley M., Ritakari T.E., Stevenson A.J., Wheater E.N.W. (2020). Translational neuroscience: The state of the nation (A PhD student perspective). Brain Commun..

[2] Chiappalone M., Semprini M. (2022). Using robots to advance clinical translation in neurorehabilitation. Sci. Robot..

[3] Bertani R., Melegari C., De Cola M.C., Bramanti A., Bramanti P., Calabrò R.S. (2017). Effects of robot-assisted upper limb rehabilitation in stroke patients: A systematic review with meta-analysis. Neurol. Sci..

[4] Charette C., Déry J., Blanchette A.K., Faure C., Routhier F., Bouyer L.J., Lamontagne M.E. (2023). A Systematic Review of the Determinants of Implementation of a Locomotor Training Program Using a Powered Exoskeleton for Individuals with a Spinal Cord Injury. Clin Rehabil..

[5] Su Y.R.S., Veeravagu A., Grant G., Laskowitz D., Grant G. (2016). Neuroplasticity after Traumatic Brain Injury. Translational Research in Traumatic Brain Injury.

[6] Nishiyama J. (2019). Plasticity of dendritic spines: Molecular function and dysfunction in neurodevelopmental disorders. Psychiatry Clin. Neurosci..

[7] Zanatta F., Farhane-Medina N.Z., Adorni R., Steca P., Giardini A., D’Addario M., Pierobon A. (2023). Combining robot-assisted therapy with virtual reality or using it alone? A systematic review on health-related quality of life in neurological patients. Health Qual. Life Outcomes.

[8] Bonanno M., De Luca R., De Nunzio A.M., Quartarone A., Calabrò R.S. (2022). Innovative Technologies in the Neurorehabilitation of Traumatic Brain Injury: A Systematic Review. Brain Sci..

[9] Prigatano G.P., Braga L.W., Johnson S.F., Souza L.M.N. (2021). Neuropsychological rehabilitation, neuroimaging and neuroplasticity: A clinical commentary. NeuroRehabilitation.

[10] Liu S., Cai W., Liu S., Zhang F., Fulham M., Feng D., Pujol S., Kikinis R. (2015). Multimodal neuroimaging computing: A review of the applications in neuropsychiatric disorders. Brain Inform..

[11] Obermeyer J.M., Ho E., Gracias A., Shoichet M.S. (2019). Influencing neuroplasticity in stroke treatment with advanced biomaterials-based approaches. Adv. Drug Deliv. Rev..

[12] Jakob V., Küderle A., Kluge F., Klucken J., Eskofier B.M., Winkler J., Winterholler M., Gassner H. (2021). Validation of a Sensor-Based Gait Analysis System with a Gold-Standard Motion Capture System in Patients with Parkinson’s Disease. Sensors.

[13] Sridhar S., Mishra S., Gulyás M., Padmanabhan P., Gulyás B. (2017). An Overview of Multimodal Neuroimaging Using Nanoprobes. Int. J. Mol. Sci..

[14] Liu R., Wang Z., Qiu S., Zhao H., Wang C., Shi X., Lin F. (2022). A Wearable Gait Analysis and Recognition Method for Parkinson’s Disease Based on Error State Kalman Filter. IEEE J. Biomed. Health Inform..

[15] Salchow-Hömmen C., Skrobot M., Jochner M.C.E., Schauer T., Kühn A.A., Wenger N. (2022). Review-Emerging Portable Technologies for Gait Analysis in Neurological Disorders. Front. Hum. Neurosci..

[16] Altimus C.M., Marlin B.J., Charalambakis N.E., Colón-Rodriquez A., Glover E.J., Izbicki P., Johnson A., Lourenco M.V., Makinson R.A., McQuail J. (2020). The Next 50 Years of Neuroscience. J. Neurosci..

[17] Andreassen O.A., Hindley G.F.L., Frei O., Smeland O.B. (2023). New insights from the last decade of research in psychiatric genetics: Discoveries, challenges and clinical implications. World Psychiatry.

[18] Toricelli M., Pereira A.A.R., Souza Abrao G., Malerba H.N., Maia J., Buck H.S., Viel T.A. (2021). Mechanisms of neuroplasticity and brain degeneration: Strategies for protection during the aging process. Neural Regen. Res..

[19] Yang T., Nie Z., Shu H., Kuang Y., Chen X., Cheng J., Yu S., Liu H. (2020). The Role of BDNF on Neural Plasticity in Depression. Front Cell Neurosci..

[20] Di Liegro C.M., Schiera G., Proia P., Di Liegro I. (2019). Physical Activity and Brain Health. Genes.

[21] Vecchio L.M., Meng Y., Xhima K., Lipsman N., Hamani C., Aubert I. (2018). The Neuroprotective Effects of Exercise: Maintaining a Healthy Brain Throughout Aging. Brain Plast..

[22] Giordano A., Clarelli F., Cannizzaro M., Mascia E., Santoro S., Sorosina M., Ferrè L., Leocani L., Esposito F. (2022). *BDNF* Val66Met Polymorphism Is Associated with Motor Recovery After Rehabilitation in Progressive Multiple Sclerosis Patients. Front. Neurol..

[23] Yu Q., Jian Z., Yang D., Zhu T. (2023). Perspective insights into hydrogels and nanomaterials for ischemic stroke. Front. Cell Neurosci..

[24] Yue W., Shen J. (2023). Local Delivery Strategies for Peptides and Proteins into the CNS: Status Quo, Challenges, and Future Perspectives. Pharmaceuticals.

[25] Dąbrowski J., Czajka A., Zielińska-Turek J., Jaroszyński J., Furtak-Niczyporuk M., Mela A., Poniatowski Ł.A., Drop B., Dorobek M., Barcikowska-Kotowicz M. (2019). Brain Functional Reserve in the Context of Neuroplasticity after Stroke. Neural Plast..

[26] Sandroff B.M., Rafizadeh C.M., Motl R.W. (2023). Neuroimaging Technology in Exercise Neurorehabilitation Research in Persons with MS: A Scoping Review. Sensors.

[27] Buxton R.B. (2013). The physics of functional magnetic resonance imaging (fMRI). Rep. Prog. Phys..

[28] Müller H.P., Kassubek J. (2013). Diffusion tensor magnetic resonance imaging in the analysis of neurodegenerative diseases. J. Vis. Exp..

[29] van Graan L.A., Lemieux L., Chaudhary U.J. (2015). Methods and utility of EEG-fMRI in epilepsy. Quant. Imaging Med. Surg..

[30] Light G.A., Williams L.E., Minow F., Sprock J., Rissling A., Sharp R., Swerdlow N.R., Braff D.L. (2010). Electroencephalography (EEG) and event-related potentials (ERPs) with human participants. Curr. Protoc. Neurosci..

[31] Maceira-Elvira P., Popa T., Schmid A.C., Hummel F.C. (2019). Wearable technology in stroke rehabilitation: Towards improved diagnosis and treatment of upper-limb motor impairment. J. Neuroeng. Rehabil..

[32] Wilson H., de Natale E.R., Politis M. (2022). Recent Advances in Neuroimaging Techniques to Assist Clinical Trials on Cell-Based Therapies in Neurodegenerative Diseases. Stem Cells..

[33] Calabrò R.S., Sorrentino G., Cassio A., Mazzoli D., Andrenelli E., Bizzarini E., Campanini I., Carmignano S.M., Cerulli S., Chisari C. (2021). Robotic-assisted gait rehabilitation following stroke: A systematic review of current guidelines and practical clinical recommendations. Eur. J. Phys. Rehabil. Med..

[34] Stampacchia G., Gazzotti V., Olivieri M., Andrenelli E., Bonaiuti D., Calabro R.S., Carmignano S.M., Cassio A., Fundaro C., Companini I. (2022). Gait robot-assisted rehabilitation in persons with spinal cord injury: A scoping review. NeuroRehabilitation.

[35] Calabrò R.S., Cacciola A., Bertè F., Manuli A., Leo A., Bramanti A., Naro A., Milardi D., Bramanti P. (2016). Robotic gait rehabilitation and substitution devices in neurological disorders: Where are we now?. Neurol. Sci..

[36] Bruni M.F., Melegari C., De Cola M.C., Bramanti A., Bramanti P., Calabrò R.S. (2018). What does best evidence tell us about robotic gait rehabilitation in stroke patients: A systematic review and meta-analysis. J. Clin. Neurosci..

[37] Liu J., Li P., Zuo S. (2023). Actuation and design innovations in earthworm-inspired soft robots: A review. Front. Bioeng. Biotechnol..

[38] Bonanno M., De Nunzio A.M., Quartarone A., Militi A., Petralito F., Calabrò R.S. (2023). Gait Analysis in Neurorehabilitation: From Research to Clinical Practice. Bioengineering.

[39] Liu K., Chen W., Yang W., Jiao Z., Yu Y. (2023). Review of the Research Progress in Soft Robots. Appl. Sci..

[40] Perpetuini D., Russo E.F., Cardone D., Palmieri R., De Giacomo A., Pellegrino R., Merla A., Calabrò R.S., Filoni S. (2023). Use and Effectiveness of Electrosuit in Neurological Disorders: A Systematic Review with Clinical Implications. Bioengineering.

[41] Pennati G.V., Bergling H., Carment L., Borg J., Lindberg P.G., Palmcrantz S. (2021). Effects of 60 Min Electrostimulation with the EXOPULSE Mollii Suit on Objective Signs of Spasticity. Front. Neurol..

[42] Lorenz M., Brade J., Diamond L., Sjölie D., Busch M., Tscheligi M., Klimant P., Heyde C.E., Hammer N. (2018). Presence and User Experience in a Virtual Environment under the Influence of Ethanol: An Explorative Study. Sci. Rep..

[43] Maggio M.G., Maresca G., De Luca R., Stagnitti M.C., Porcari B., Ferrera M.C., Galletti F., Casella C., Manuli A., Calabrò R.S. (2019). The Growing Use of Virtual Reality in Cognitive Rehabilitation: Fact, Fake or Vision? A Scoping Review. J. Natl. Med. Assoc..

[44] De Luca R., Bonanno M., Rifici C., Pollicino P., Caminiti A., Morone G., Calabrò R.S. (2022). Does Non-Immersive Virtual Reality Improve Attention Processes in Severe Traumatic Brain Injury? Encouraging Data from a Pilot Study. Brain Sci..

[45] De Luca R., Bonanno M., Marra A., Rifici C., Pollicino P., Caminiti A., Castorina M.V., Santamato A., Quartarone A., Calabrò R.S. (2023). Can Virtual Reality Cognitive Rehabilitation Improve Executive Functioning and Coping Strategies in Traumatic Brain Injury? A Pilot Study. Brain Sci..

[46] Varela-Aldás J., Buele J., Ramos Lorente P., García-Magariño I., Palacios-Navarro G. (2021). A Virtual Reality-Based Cognitive Telerehabilitation System for Use in the COVID-19 Pandemic. Sustainability.

[47] Goffredo M., Pagliari C., Turolla A., Tassorelli C., Di Tella S., Federico S., Pournajaf S., Jonsdottir J., De Icco R., Pellicciari L. (2023). Non-Immersive Virtual Reality Telerehabilitation System Improves Postural Balance in People with Chronic Neurological Diseases. J. Clin. Med..

[48] Liew S.L., Santarnecchi E., Buch E.R., Cohen L.G. (2014). Non-invasive brain stimulation in neurorehabilitation: Local and distant effects for motor recovery. Front. Hum. Neurosci..

[49] Li K.P., Wu J.J., Zhou Z.L., Xu D.S., Zheng M.X., Hua X.Y., Xu J.G. (2023). Noninvasive Brain Stimulation for Neurorehabilitation in Post-Stroke Patients. Brain Sci..

[50] Banduni O., Saini M., Singh N., Nath D., Kumaran S.S., Kumar N., Srivastava M.V.P., Mehndiratta A. (2023). Post-Stroke Rehabilitation of Distal Upper Limb with New Perspective Technologies: Virtual Reality and Repetitive Transcranial Magnetic Stimulation—A Mini Review. J. Clin. Med..

[51] Groiss S.J., Wojtecki L., Südmeyer M., Schnitzler A. (2009). Deep brain stimulation in Parkinson’s disease. Ther. Adv. Neurol. Disord..

[52] Zanos S. (2019). Closed-Loop Neuromodulation in Physiological and Translational Research. Cold Spring Harb. Perspect. Med..

[53] Shah R.S., Chang S.Y., Min H.K., Cho Z.H., Blaha C.D., Lee K.H. (2010). Deep brain stimulation: Technology at the cutting edge. J. Clin. Neurol..

[54] Haug C.J., Drazen J.M. (2023). Artificial Intelligence and Machine Learning in Clinical Medicine. N. Engl. J. Med..

[55] Erickson B.J. (2021). Basic Artificial Intelligence Techniques: Machine Learning and Deep Learning. Radiol. Clin. N. Am..

[56] Alsobhi M., Sachdev H.S., Chevidikunnan M.F., Basuodan R., K U D.K., Khan F. (2022). Facilitators and Barriers of Artificial Intelligence Applications in Rehabilitation: A Mixed-Method Approach. Int. J. Environ. Res. Public Health.

[57] Kristoffersson A., Lindén M. (2022). A Systematic Review of Wearable Sensors for Monitoring Physical Activity. Sensors.

[58] Cecil C.A.M., Nigg J.T. (2022). Epigenetics and ADHD: Reflections on Current Knowledge, Research Priorities and Translational Potential. Mol. Diagn. Ther..

[59] Segato A., Marzullo A., Calimeri F., De Momi E. (2020). Artificial intelligence for brain diseases: A systematic review. APL Bioeng..

[60] Chen Z.S., Kulkarni P.P., Galatzer-Levy I.R., Bigio B., Nasca C., Zhang Y. (2022). Modern views of machine learning for precision psychiatry. Patterns.

[61] Zhang X., Yao L., Wang X., Monaghan J., McAlpine D., Zhang Y. (2021). A survey on deep learning-based non-invasive brain signals: Recent advances and new frontiers. J. Neural. Eng..

[62] Zhang Y., Naparstek S., Gordon J., Watts M., Shpigel E., El-Said D., Badami F.S., Eisenberg M.L., Toll R.T., Gage A. (2023). Machine learning-based identification of a psychotherapy-predictive electroencephalographic signature in PTSD. Nat. Ment. Health.

[63] Preatoni E., Nodari S., Lopomo N.F. (2020). Supervised Machine Learning Applied to Wearable Sensor Data Can Accurately Classify Functional Fitness Exercises within a Continuous Workout. Front. Bioeng. Biotechnol..

[64] Slemenšek J., Fister I., Geršak J., Bratina B., van Midden V.M., Pirtošek Z., Šafarič R. (2023). Human Gait Activity Recognition Machine Learning Methods. Sensors.

[65] Siegelmann H.T. (2010). Complex systems science and brain dynamics. Front. Comput. Neurosci..

[66] Kenzie E.S., Parks E.L., Carney N., Wakeland W. (2022). System dynamics modeling for traumatic brain injury: Mini-review of applications. Front. Bioeng. Biotechnol..

[67] Hernandez L.M., Blazer D.G., Institute of Medicine (US) Committee on Assessing Interactions among Social, Behavioral, and Genetic Factors in Health (2006). Genes, Behavior, and the Social Environment: Moving Beyond the Nature/Nurture Debate.

[68] Garro F., Chiappalone M., Buccelli S., De Michieli L., Semprini M. (2021). Neuromechanical Biomarkers for Robotic Neurorehabilitation. Front. Neurorobot..

[69] Lo K., Stephenson M., Lockwood C. (2019). The economic cost of robotic rehabilitation for adult stroke patients: A systematic review. JBI Database Syst. Rev. Implement Rep..

[70] Ciesielski T.H., Aldrich M.C., Marsit C.J., Hiatt R.A., Williams S.M. (2017). Transdisciplinary approaches enhance the production of translational knowledge. Transl Res..

